# The Poor-Law Midwifery Schools

**Published:** 1908-02-08

**Authors:** 


### Nursing Administration.

### THE POOR-LAW MIDWIFERY SCHOOLS.

### THE VALUE OF SMALL CENTRES.

In previous articles it has been shown that, while the Central Midwives Board must be completely exonerated from the charge of wilfully refusing to recognise really satisfactory midwifery training schools in Poor-law establishments, yet that the effect of their policy has been, through needless secrecy, to discourage eligible applications and to rouse an expectation of being recognised in many institutions which are ineligible for lack of material for instruction. The Board has undoubtedly shown grave lack of business methods in refusing to make public the principles which guides it in acceptance or rejection of institutions as recognised training centres. It is greatly to be regretted that the same policy is being observed in respect of the recognition of medical officers as teachers of midwifery. The rules of the Central Midwives Board in regard to the preparation of candidates for examination state that the certificates as to the practical midwifery work undertaken by the candidate must be filled up and signed either by a medical registered practitioner or by the chief midwife, or, in the absence of such an officer, by the matron of an institution recognised by the Board, or, in the case of Poor-law institutions, by the matron, being a midwife certified under the Midwives Act, or a superintendent nurse . . . or a midwife certified under the Act, and approved by the Board for that purpose.

In addition to this practical work the candidate must have attended a three months' course of instruction in theoretical midwifery, and present a certificate to this effect, filled up and signed by a registered medical practitioner, recognised by the Board as a teacher.

The result of this regulation is to place serious difficulties in the way of candidates being prepared for examination in small centres after private study. It will be observed that the Board lays down no qualification which should entitle a "registered medical practitioner" to give lessons in midwifery to pupil midwives. In the case of the Bethnal Green Infirmary we learn that the Guardians were informed by the Board that "if the Medical Officer of the Infirmary (a London M.D. gold medallist) chose to apply for recognition as a teacher, his application would be considered." Now, it may be indolence or it may be haughtiness, but it appears the medical practitioner does not "choose to apply" for permission to consider himself qualified to teach the elements of midwifery to a few young women and expose himself to the chance of a blank refusal. The Board is clearly guided by some general principle in recognising teachers of midwifery. Let it state this principle clearly, and declare what qualifications are essential, and then let all who possess these qualifications be recognised as a matter of course without any fresh filling up of forms or "consideration by the Board." By this means a very large access of well-trained candidates for examination could be speedily secured. For at the present moment there is in many excellent establishments a great waste of material for training midwives on account of the difficulty of getting courses of instruction by recognised teachers. The instruction given to one or two pupils privately is likely to be far more efficacious in bringing them to an understanding of the difficulties in their work than any series of lectures to large numbers. There is hardly a town of any size throughout the country where, under the supervision of highly-trained nurses the practical work necessary to qualify for examination could not be obtained. The only bar to utilising this mass of material is the want of recognised teachers. And so long as the qualifications which admit medical men to this sacred band of privileged teachers are kept shrouded in darkness, the number is not likely to increase very rapidly. At present the usual means of fitting women to undertake midwifery work in the rural districts is to raise from fifteen to twenty pounds and send them to be trained in London. The result is very often to unsettle them permanently for the very work they are intended to pursue. It would be vastly better that such women should serve an apprenticeship in their own county under a midwife qualified to give instruction, and we believe that were the door of "recognition" set open instead of being tight shut, with no visible latch, medical men would make no difficulties about preparing the candidates for examination.

The Central Midwives Board has had to grapple with an immense mass of business and to create an entirely new department, with no precedents to guide its actions. It has been hampered in scores of ways, and has been compelled to make its way in the teeth of much public distrust and private hostility. It is too early yet to pronounce upon the effect of the Act which brought the Board into being. But the courageous manner in which the members have grappled with the arduous duties entrusted to them by Parliament has roused general admiration, and the body of work already accomplished marks a great achievement in the history of midwifery in this country. Enough has been done to make it clear that the Board can count on the support of all who understand the weighty issues involved in creating and maintaining a high standard of midwifery. But we look now for a more forward policy. Large new training centres for midwives are neither to be expected or desired. But in whatever spot the required number of births takes place, from one end of the country to another, here is a possible centre of instruction. It remains for the Board to see to it that all the available material for training candidates is used to the utmost, and to this end we are confident nothing will conduce but a policy of frank encouragement, in place of secrecy and distrust.

